# Artificial Neural Network to Predict Varicocele Impact on Male Fertility through Testicular Endocannabinoid Gene Expression Profiles

**DOI:** 10.1155/2018/3591086

**Published:** 2018-11-13

**Authors:** Davide Perruzza, Nicola Bernabò, Cinzia Rapino, Luca Valbonetti, Ilaria Falanga, Valentina Russo, Annunziata Mauro, Paolo Berardinelli, Liborio Stuppia, Mauro Maccarrone, Barbara Barboni

**Affiliations:** ^1^Faculty of Biosciences and Technology for Food, Agriculture and Environment, University of Teramo, 64100 Teramo, Italy; ^2^Faculty of Veterinary Medicine, Agriculture and Environment, University of Teramo, 64100 Teramo, Italy; ^3^Department of Psychological, Health and Territorial Sciences, School of Medicine and Health Sciences, University “G. d'Annunzio” of Chieti and Pescara, 66100 Chieti, Italy; ^4^Department of Medicine, Campus Bio-Medico University of Rome, 00128 Rome, Italy; ^5^European Center for Brain Research, IRCCS Santa Lucia Foundation, 00164 Rome, Italy

## Abstract

The relationship between varicocele and fertility has always been a matter of debate because of the absence of predictive clinical indicators or molecular markers able to define the severity of this disease. Even though accumulated evidence demonstrated that the endocannabinoid system (ECS) plays a central role in male reproductive biology, particularly in the testicular compartment, to date no data point to a role for ECS in the etiopathogenesis of varicocele. Therefore, the present research has been designed to investigate the relationship between testicular ECS gene expression and fertility, using a validated animal model of experimental varicocele (VAR), taking advantage of traditional statistical approaches and artificial neural network (ANN). Experimental induction of VAR led to a clear reduction of spermatogenesis in left testes ranging from a mild (Johnsen score 7: 21%) to a severe (Johnsen score 4: 58%) damage of the germinal epithelium. However, the mean number of new-borns recorded after two sequential matings was quite variable and independent of the Johnsen score. While the gene expression of biosynthetic and degrading enzymes of AEA (NAPE-PLD and FAAH, respectively) and of 2-AG (DAGL*α* and MAGL, respectively), as well as their binding cannabinoid receptors (CB_1_ and CB_2_), did not change between testes and among groups, a significant downregulation of vanilloid (TRPV1) expression was recorded in left testes of VAR rats and positively correlated with animal fertility. Interestingly, an ANN trained by inserting the left and right testicular ECS gene expression profiles (inputs) was able to predict varicocele impact on male fertility in terms of mean number of new-borns delivered (outputs), with a very high accuracy (average prediction error of 1%). The present study provides unprecedented information on testicular ECS gene expression patterns during varicocele, by developing a freely available predictive ANN model that may open new perspectives in the diagnosis of varicocele-associated infertility.

## 1. Introduction

Varicocele is considered among the most common causes of male infertility and affects approximately 15-20% of the general male population. It is reported in 19-41% of men with primary infertility, as well as in up to 80% of men with secondary infertility [1]. Since the first evidence supporting a link between varicocele and infertility was published [2, 3], an extensive research effort has been addressed to elucidate underling mechanisms and their relationship with varicocele-associated testicular dysfunction and male fertility outcome.

Varicocele is functionally related to an abnormal dilation and tortuosity of the pampiniform plexus veins, which are responsible for the testicular venous drainage. This defect is believed to be the main trigger of varicocele, inducing a complex and multifactorial cascade of events that may synergistically lead to infertility and impair spermatogenesis [4–6]. Among others, scrotal hyperthermia, testicular hypoperfusion and hypoxia, oxidative stress, endocrine and paracrine disturbances, backflow of adrenal metabolites, genetic disorders, and autoimmunity can all be detrimental factors for testicular function, participating in varicocele-related infertility mechanisms [1, 7–10].

Clinically, up to 90% of varicocele diagnoses refer to a unilateral, left-sided disease [10]. Varicocele is well-known to be associated with poorer semen quality and infertility, reduced testicular volume, decline of Leydig cell secretion, and altered reproductive hormone levels, such as follicle stimulating hormone (FSH), inhibin B, and luteinizing hormone (LH) [11–13]. As a consequence of the unbalance between local and systemic reproductive hormones, an increased apoptosis of germ cells [14], as well as impairment of their maturation with the emergence of epigenetic disorders [15, 16], is frequently triggered.

Varicocele can express different degrees of severity: according to Dubin and Amelar [17]* grade I* recognizes a palpable defect only during Valsalva manoeuvre,* grade II* a palpable deficit in the upright position, and* grade III* a disease visible also without any palpation. Instead,* subclinical* varicoceles are all the cases where the pathology can be only detectable by ultrasound, angiography, and other imaging techniques [18].

Nonetheless, the clinical severity of the disease is not always related to the quality of semen samples: indeed, 45-65% of men with grades 1-3 varicoceles report no alterations of semen parameters [13]. In addition, varicocele has frequently confounding medical conditions with respect to the impact on fertility [19]. Even though approximately 80% of men with varicocele preserve their fecundity [20, 21], the disease can be alternatively associated with severe infertility. Furthermore, on the basis of available evidence it remains rather difficult to make any relationship between semen data and fertility outcome. Indeed, varicocele can be found in men with both abnormal and normal seminal parameters, without differences in the spontaneous pregnancy rate [11]. For this reason, the improvement in predictivity of varicocele diagnostic procedures remains an open clinical challenge.

Over the years, a conspicuous amount of studies has been carried out identifying several new bioactive molecules in reproduction. Among them, a key role in female and male reproductive physiology has been recognized for the endocannabinoid system (ECS) [22–27]. ECS consists of G-protein coupled type 1 and type 2 cannabinoid (CB_1_ and CB_2_) receptors; endogenous ligands of such receptors, known as endocannabinoids (eCBs) [mainly anandamide (*N*-arachidonoylethanolamine) and 2-AG (2-arachidonoylglycerol)]; and enzymes regulating the biosynthesis [i.e.,* N*-acylphosphatidylethanolamine-selective phospholipase D (NAPE-PLD) and diacylglycerol lipases (DAGL) *α* and *β*, for AEA and 2-AG, respectively] and inactivation of eCBs [i.e., fatty acid amide hydrolase (FAAH) and monoacylglycerol lipase (MAGL), for AEA and 2-AG, respectively] [28, 29]. Furthermore, accumulated evidence suggests the presence of additional receptor targets for eCBs on the cell surface, such as the transient receptor potential vanilloid 1 (TRPV1) ion channel [27, 30]. ECS in male reproduction has been described as a conserved system acting from sea urchin to humans [31–34] through the control of several mechanisms related to the physiology of male germ cells and testicular somatic cells as well [35–38]. In humans, ECS involvement in regulating reproductive pathways has been strongly supported since the identification of an active ECS in reproductive fluids [39]. Notably, eCBs and their relative receptors are present along the whole hypothalamic-pituitary-gonadal (HPG) axis and, at central level, it is a consensus opinion that eCBs negatively modulate gonadotropin discharge by inhibiting the release of hypothalamic gonadotropin-releasing hormone (GnRH) [40, 41]. Indeed, in mediobasal hypothalamus, intracerebroventricular injection of AEA suppresses GnRH release [42], and altered GnRH signalling was documented in CB_1_^–/–^ mice [43]. Furthermore, 2-AG suppresses LH secretion in wild-type but not in CB_1_^–/–^ mice, whereas AEA decreases LH levels also in CB_1_^–/–^ [44], meaning that receptors other than CB_1_, i.e., TRPV1, might participate in such a modulation. At gonadal level, an intricate crosstalk between eCBs and reproductive hormones is involved in the control of several activities. A significant reduction of serum LH and testosterone, as well as lower* in vitro* basal secretion of testosterone, have been noticed in CB_1_^–/–^ mice, with AEA suppressing LH and testosterone secretion in wild-type mice but not in knockout animals [45]. Moreover, the presence of CB_1_ in Leydig cells and its leading role in testosterone secretion confirm the importance of eCBs in modulating Leydig cells functions and testis endocrinology [41, 46]. Particularly, CB_1_ expression in differentiating rat adult Leydig cells is also negatively correlated with cell proliferation, and the presence of few adult Leydig cells in CB_1_^–/–^ mice points to the ECS as a potential modulator of proliferative activity involved in adult Leydig cells differentiation [47] and may also explain the lower in vitro basal testosterone secretion in CB_1_^–/–^ mice [45]. Furthermore, a pivotal role of CB_2_ in mouse spermatogenesis has also been postulated [48]. On the basis of decreasing levels of 2-AG detected from mice spermatogonia to spermatocytes and spermatids, it is likely to argue that 2-AG, through CB_2_-signaling, may act as an autocrine/paracrine mediator in controlling and promoting spermatogonia progression into meiosis. Conversely, testicular AEA needs to be maintained at an appropriate locally tone in order to allow normal spermatogenesis progression [48]. In support of this observation, alterations in eCB signaling, by* in vivo *administration of a potent agonist of CB receptors, cause a marked impairment of spermatogenesis in rat testes [49]. Moreover, genetic inactivation of CB_1_ causes an inefficient histone displacement, poor chromatin condensation, and DNA damage in mouse spermatids, demonstrating the involvement of ECS-mediated epigenetic mechanisms driving spermiogenesis [50]. Of note, AEA induces DNA fragmentation and apoptosis in Sertoli cells, by a mechanism reversed by FSH through PKA and aromatase-dependent activation of fatty acid amide hydrolase (FAAH), via epigenetic regulation of gene expression [51, 52].

Despite accumulated evidence on the influence of ECS on testis homeostasis, no data are yet available on the possible role of ECS during varicocele pathogenesis. Therefore, the present study has been designed to detect ECS gene expression profiles during the early phase of varicocele by using a validated experimental model of the disease in rats. ECS gene expression profiles were evaluated in both testes and then were related to the reproductive outcomes defined as the mean number of new-borns recorded after two consecutive mating cycles, once varicocele was established. The relationship between ECS gene expression profiles and fertility outcomes were then analyzed by building up reliable statistical approaches. Probably due to the complexity of reproductive pathways, either the univariate or the multiple regression analysis tests failed to show any consistent correlation. Instead, the analytic limits of the previous traditional testing were overcome by adopting an artificial neural network (ANN). The ANN was developed by using as inputs the ECS gene patterns in both the testes and as outputs the mean number of new-borns. Once trained, the ANN was able to predict with a very high degree of accuracy (99%) the male fertility, starting by the altered ECS gene expression profiles induced by the experimental varicocele. Therefore, ANN offers a new, robust, and freely available diagnostic clinical tool to trace the reproductive prognosis of varicocele, thus facing this confounding medical condition by predicting its impact on fertility.

## 2. Materials and Methods

### 2.1. Animals

Male Sprague-Dawley rats were housed at a temperature of 21 ± 2°C, with a humidity percentage of 55 ± 10%, and maintained under a 12-hour light-dark cycle. The animals were fed with a standard pellet diet and water* ad libitum* for 6 to 8 weeks (300-400g) before receiving surgical manoeuvres.

### 2.2. Experimental Design and Surgery

**Figure 1:**
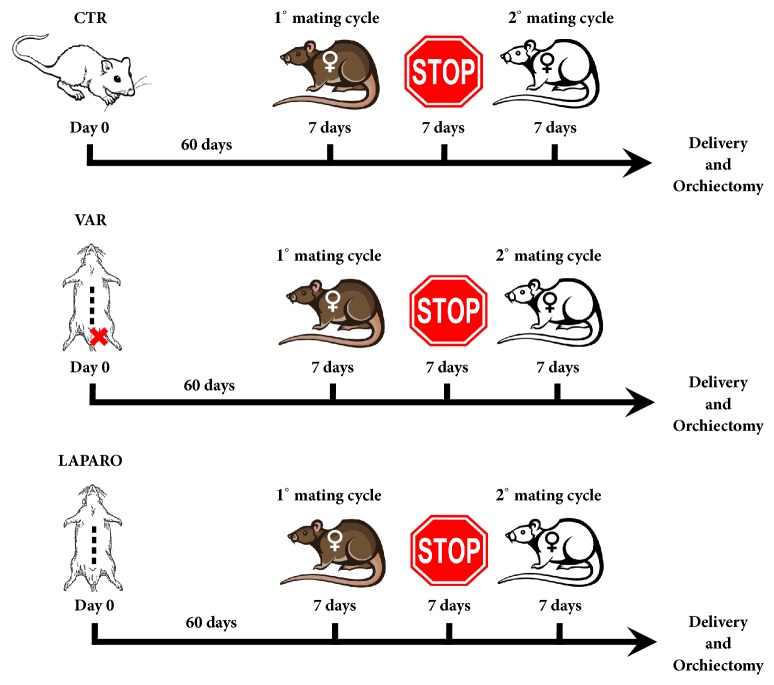
**Experimental design**. In this figure the different experimental groups are summarized: CTR, healthy animals; VAR, surgical induced varicocele rats; LAPARO, animals only subjected to a surgical laparotomy approach. To assess the male reproductive outcomes, animals belonging to the experimental groups, after sixty days, were bred with two different females of proved fertility in order to promote two consecutive mating cycles. Each mating cycle lasted 7 days and was spaced by 7 days of reproductive stop. The experimental animals were sacrificed and bilateral orchiectomies were performed when both the broods were delivered, and the number of new-borns were recorded.

All the experimental procedures were authorized by Italian Ministry of Health (approval ID 301/2015-PR 27/04/2015). A total of 35 animals were randomly divided into three groups as summarized in Figure 1: control (CTR;* n *= 6), laparotomy (LAPARO,* n *= 5), and varicocele (VAR;* n *= 24).

The surgical induction of varicocele was performed according to Turner [53]. In detail, after general anaesthesia with intraperitoneal injection of 30-60 mg/kg Pentothal Sodium, the upper left abdominal quadrant was approached through a midline laparotomy incision. The abdominal contents were packed to the right in order to visualize the left kidney, the left adrenal vein, the left renal vein, and the left spermatic vein as it inserts into the left renal vein. A 4-0 silk suture was used to partially occlude the left renal vein upstream of the confluence with the left spermatic vein. This occlusion increased the intravenous pressure lateral to the obstruction, and the pressure was transmitted to the left spermatic vein causing a varicocele to develop. The animals belonging to LAPARO group received exclusively the midline laparotomy incision, in order to assess whether this surgical manoeuvre might have an impact on the male mating behaviour. Finally, CTR group is represented by healthy animals.

To explore the impact of experimental models (VAR and LAPARO) upon fertility, the animals were mated after 60 days from the surgical procedures. During this window, since spermatogenesis in Sprague-Dawley rats lasts approximately 51.6 days [54], each rat completed one wave of spermatogenesis, while the varicocele dysfunction was established. After these 60 days, all rats were bred consecutively in the presence of two different females of proved fertility, in order to promote mating. The reproductive outcome in terms of new-borns was recorded after both matings. Then, after the second brood was delivered, the experimental animals were sacrificed in order to explant both testes (Figure 1). Testis samples were divided into halves and subjected each to morphological and molecular detections.

### 2.3. Histopathological Analysis

The histological analysis was carried out on a total of 30 animals (CTR* n *= 6; LAPARO* n* = 5; VAR* n* = 19) and performed on both testes. Testicular explants were fixed overnight at 4°C in paraformaldehyde solution 4% in PBS. Five-micron thick sections, obtained from paraffin-embedded tissues, were then stained with hematoxylin and eosin (H&E). The histopathological changes of the testes were evaluated with an Axioskop 2 Plus light microscope (Zeiss), grading the seminiferous tubules according to the Johnsen scoring system [55]. In detail, the different scores were blinded assigned by adopting following morphological criteria:

- 10: complete spermatogenesis with many spermatozoa, germinal epithelium organized in a regular thickness leaving an open lumen
- 9: many spermatozoa present but disorganized germinal epithelium with marked sloughing or obliteration of lumen
- 8: only few spermatozoa present
- 7: no spermatozoa but many spermatids present
- 6: no spermatozoa and only few spermatids present
- 5: no spermatozoa, no spermatids but several or many spermatocytes present
- 4: only few spermatocytes and no spermatids or spermatozoa present
- 3: only spermatogonia present
- 2: no germ cells but Sertoli cells are present
- 1: no cells in tubular section

The Johnsen score of each testis was calculated as the mean value from at least ten randomly selected seminiferous tubules.

### 2.4. Quantitative Real Time-Reverse Transcriptase-Polymerase Chain Reaction (qRT-PCR) Analysis

Total RNA was extracted from testicular samples by using TRIzol (Life technologies, Grand Island, NY) according to the manufacturer instructions. Quantification of RNA samples was performed by using Thermo Scientific NanoDrop 2000c UV-Vis spectrophotometer at 260 nm (Waltham, MA USA). Contaminating DNA was digested by treating RNA samples with DNase I (Sigma-Aldrich, Darmstadt, Germany) for 15 min at RT. cDNA was synthetized from 1 *μ*g of total RNA of each sample by using the RevertAid H Minus First Strand cDNA Synthesis Kit (Thermo Scientific, Waltham, MA, USA). The relative abundance was assessed by RT quantitative PCR (RT-qPCR) using SensiFAST™ SYBR Lo-ROX kit (Bioline, London, UK) by adjusting the manufacturer instruction to a final volume of 15 *μ*L on a 7500 Fast Real-Time PCR System (Life Technologies, Grand Island, NY). To provide precise quantification of the initial target in each PCR reaction, the amplification plot was examined, as well as the point of early log phase of product accumulation defined by assigning a fluorescence threshold above background, defined as the threshold cycle number or Ct. The relative expression of different amplicons was calculated by the delta–delta Ct (ΔΔCt) method and converted to relative expression ratio (2^-ΔΔCt^) for statistical analysis [56]. All data were normalized to the endogenous reference gene GAPDH. The primer used for PCR amplification is reported in Table 1.

### 2.5. Data Analysis

The data analyzed in this study were the left and right testes Johnsen scores, the male reproductive outcomes expressed as the mean number of new-borns delivered in two consecutive mating cycles, and the expression of each ECS target gene as the respective 2^-ΔΔCt^ value.

Attempting to model the relationship between left testicular histopathological scores (Johnsen scores) and reproductive outcomes, a regression analysis was performed (Excel 2010).

D'Agostino-Pearson normality test was used to check the data for normal distribution. One-way ANOVA followed by* post hoc* Turkey's multiple comparison test and Kruskal-Wallis with* post hoc* Dunn's multiple comparison test was used as appropriate;* p*-values <0.05 were considered statistically significant (GraphPad Prism 6). Different models were used to analyze and relate the ECS gene expression data to the reproductive outcomes. First, the Pearson correlation coefficient *r* among all the variables was checked, followed by a multiple regression analysis calculating multiple R and R^2^ (Excel 2010, Past3). Finally, an Artificial Neural Network (ANN) was made using JustNN. The mean number of pups as reproductive output was set up together with the ECS gene expression profiles considered as the biological input values. The network had a growth rate of 10 cycles or 5 seconds, 1 hidden layer, and a learning rate of 0.6. The target error was fixed <0.01, one hundred cycles before validating cycle and 100 cycles per validating cycle were used, and the learning process was stopped when all the validating examples were within the 10% as validating error.

## 3. Results

### 3.1. Correlation between Testicular Morphology and Fertility

To determine the impact of the surgical procedures on testicular microarchitecture, histological evaluations were carried out on paraffin-embedded sections of both the testes.

**Figure 2:**
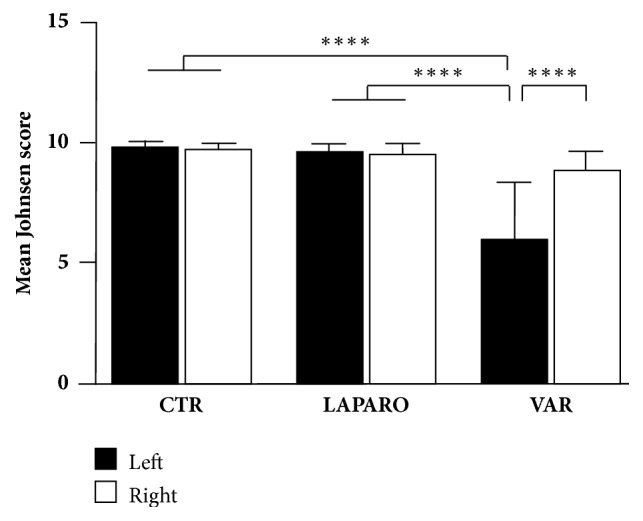
**Johnsen score analysis of testicular explants. **In VAR group, a significant decrease of the mean Johnsen score value was recorded in the left testes compared to contralateral and CTR/LAPARO left and right testes. One-way ANOVA followed by Turkey's post hoc test was performed; data were expressed as mean with SD; *∗∗∗∗p*<0.0001.

The surgical induction of pathology elicited a significant reduction of the mean Johnsen score values recoded in left testes of VAR group (*p *< 0.0001, VAR left versus CTR;* p *< 0.0001, VAR left versus LAPARO;* p *< 0.0001, VAR left versus VAR right). The morphological analysis, in addition, demonstrated that after approximately 120 days from the surgical procedures, the right testes of VAR rats did not display any compensatory adaptation. Indeed, they showed a preserved tissue microarchitecture with only a slight decrease in the Johnsen mean score values. On the contrary, LAPARO group animals did not show any tissue defects on both testes (Figure 2).

**Figure 3:**
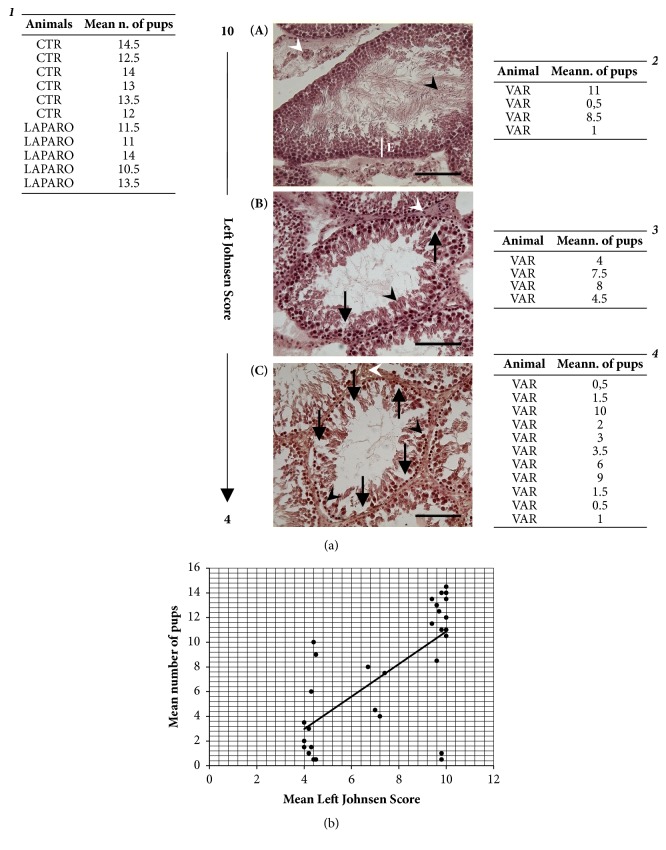
**Morphological analysis and correlation with fertility outcomes**. A total of 30 animals (CTR* n *= 6; LAPARO* n* = 5; VAR* n* = 19) were subjected to the morphological evaluation. Panel (a): (**A**-**C**) example photomicrographs of left testes sections stained with H&E. (***1-4***) Tables reporting the mean number of pups recorded in experimental animals displaying different Johnsen scores. (**A**) Animals with a Johnsen score of 10 showed a complete spermatogenesis with many spermatozoa (black arrow head), and the seminiferous epithelium (E) organized in a regular thickness (white line); polyhedral Leydig cells with a round nucleus and eosinophilic cytoplasm were visible in interstitial compartments (white arrow head). (**B**) Samples with a Johnsen score of 7 displayed a mild impairment of the spermatogenic process with many spermatids (black arrow head) and some disorganization foci (black arrows) in the germinal epithelium; Leydig cells with smaller nuclei and accumulated connective tissue were also detectable in some interstitia (white arrow head). (**C**) Animals with a Johnsen score of 4 reported a compromised spermatogenesis with only few spermatocytes (black arrow heads) present and a damaged seminipherous epithelium with many disorganization foci (black arrows); interstitial compartments were not always conserved and also displayed uneven Leydig cells with not clearly identifiable cellular edges (white arrow head). Scale bars = 100 *μ*m. (***1***) A conserved reproductive outcome was recorded in LAPARO group which showed, as CTR group, a complete spermatogenesis. (***2***) A conserved spermatogenesis was also recorded in four VAR animals showing a quite variable mean number of pups. (***3***) A mild impairment of the spermatogenic process was recorded in four VAR animals with a mean number of pups ranging from 4 to 8. (***4***) The majority of VAR animals (11) displayed a compromised spermatogenesis with a fertility outcome ranging from 0.5 to 10 new-borns. Panel (b): regression analysis of the mean Johnsen score values recorded in left testes versus the mean reproductive outcome; R^2^ = 0,465.

More in detail, the analyses of tissue sections obtained from the left testes revealed that all the animals of both CTR and LAPARO (11 out of 11) displayed groups of seminiferous tubules with several spermatozoa and a germinal epithelium organized in a regular thickness leaving an open lumen (Johnsen score of 10). Conversely, different degrees of damage were recorded in the VAR animals (Figure 3(a)). In particular, the H&E staining of the VAR samples showed left testes with Johnsen scores ranging from 10 to 4. A normal organization of the germinal epithelium and a complete spermatogenesis were recorded in 21% of studied animals (Johnsen score 10, Figure 3(A)). Another 21% showed a not fully organized germinal epithelium with no spermatozoa and many late spermatids (Johnsen score 7, Figure 3(B)). The majority of the testes (58%) displayed a wide-spread impairment of the germinal epithelium with no spermatozoa or spermatids and few spermatocytes present (Johnsen score 4, Figure 3(C)).

As summarized in Figure 3(a), Tables* 1*-*4*, the Johnsen score values detected in left testes did not appear predictive of the male fertility outcome. Indeed, the mean number of new-borns recorded after two sequential matings was quite variable and independent of the testis organization degree. A drastic fertility reduction was observed even in VAR group animals reporting a high Johnsen score, and a high number of pups were recorded also in animals with a clearly damaged testis (Johnsen score of 4).

The absence of a consistent relationship between varicocele-induced tissue damage and fertility was clearly confirmed by the regression analysis performed by using the Johnsen score of the left testes as the explanatory variable of the mean number of pups. The regression analysis resulted in R^2^ = 0.465, and the scatter plot highlighted that an increasing Johnsen score value did not always match with an increased fertility, while a decreasing Johnsen score value did not always witness a decreased reproductive outcome, thus demonstrating that a linear model cannot satisfactorily explain the relationship between varicocele-induced testicular damages and male fertility (Figure 3(b)).

### 3.2. ECS Gene Expression

Since LAPARO procedures did not affect either testicular morphology or fertility output, rats belonging to CTR and LAPARO groups were pooled (*n *= 11) and altogether considered as CTRL animals in the subsequent experiments and analyses.

**Figure 4:**
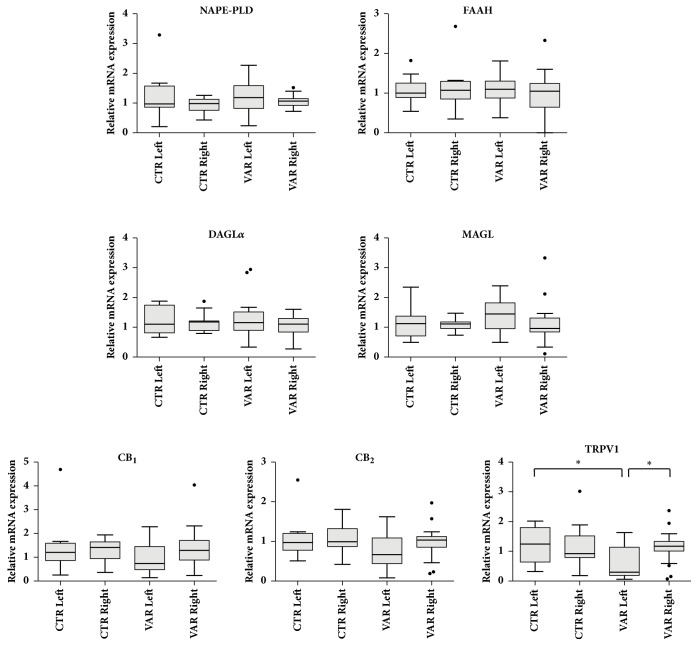
**Effect of experimental varicocele on testicular ECS gene expression**. qRT-PCR analysis of the main ECS components (i.e., NAPE-PLD, DAGL*α*, FAAH, MAGL, CB_1_, CB_2_, and TRPV1) in left and right testes of different groups: CTRL (*n *= 11; CTR and LAPARO animals pooled) and VAR (*n* = 24). Data were expressed as Turkey-style box plot and whiskers and analyzed by Kruskal-Wallis followed by* post hoc* Dunn's multiple comparison test; *∗p* < 0.05.

Gene expression of the main components of ECS has been investigated in both left and right testes of CTRL and VAR animals, by means of qRT-PCR (Figure 4). First, the presence of metabolizing enzymes of AEA (NAPE-PLD and FAAH, respectively) and 2-AG (DAGL*α* and MAGL; respectively), as well as cannabinoid (CB_1_ and CB_2_) and vanilloid (TRPV1) receptors, has been confirmed at gene level in CTRL group, and no statistically significant differences were found between left and right testes. Interestingly, a significant decrease in TRPV1 mRNA expression was reported in VAR left testes with respect to CTRL left and VAR right ones (both* p*<0.05) (Figure 4). Noteworthy, none of the other ECS elements investigated (i.e., NAPE-PLD, DAGL*α*, FAAH, MAGL, CB_1_, and CB_2_) was affected by varicocele induction.

### 3.3. Correlation between ECS Gene Expression and Fertility Using a Univariate Statistical Analysis

In order to investigate a potential relationship between testicular ECS gene expression levels and reproductive outcomes in CTRL and VAR groups, a univariate correlation analysis was performed. In particular, the Pearson correlation coefficients and* p* values were calculated (Table 2). The variables considered for this analysis were 2^-ΔΔCt^ values of each ECS target gene (NAPE-PLD, FAAH, DAGL*α*, MAGL, CB1, CB2, and TRPV1) in both left and right testes and the mean reproductive performances. The analysis demonstrated a positive correlation between TRPV1 mRNA level in left testes and reproductive outcomes (*r* = 0.383 and* p* = 0.023). No other correlations were found among the other ECS genes and reproductive outputs.

However, a number of statistically significant correlations were observed among ECS components, as shown by *r* and *p* coefficients reported in Table 2.

### 3.4. Correlation between ECS Gene Expression and Fertility Using a Multiple Regression Model

**Figure 5:**
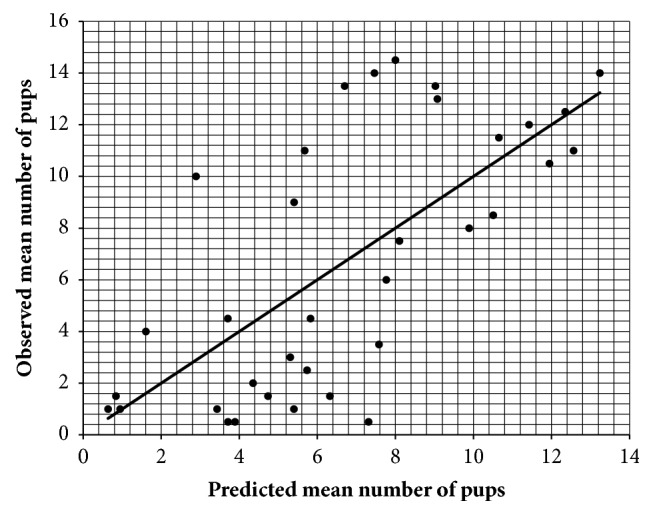
**Multiple regression model**. Multiple regression analysis among the ECS gene expression levels recorded in both left and right testes and the mean number of pups; multiple R = 0.693 and R^2^ = 0.480.

Since the poor relationship between ECS gene expression and fertility may be the consequence of the biological complexity of the processes studied, other statistical approaches have been attempted. Incidentally, most of the events that lead to the achievement of fertility are characterized by the presence of cooperative elements that make the system controlled by feedbacks, self-loops, and nonlinear interactions. Based on this evidence, a multiple regression analysis was performed. Specifically, 2^-ΔΔCt^ values of each ECS target gene under investigation, in both left and right testes, were used as explanatory variables of the mean number of pups. By using this statistical approach, the predictability of the system's output was increased, resulting in multiple R and R^2^ values of 0.693 and 0.480, respectively. In particular, the data were symmetrically dispersed with respect to the line representing the best data fitting, thus demonstrating that 48% of the variance in reproductive outcomes can be explained and predicted using ECS gene expression profiles in the left and right testes (Figure 5).

### 3.5. Artificial Neural Network (ANN) to Analyze the Relationship between ECS Gene Expression and Male Fertility

From a biological point of view, even if the model reliability greatly increased passing from a univariate correlation to a multiple regression approach, this latter still remained unsatisfactory.

**Figure 6:**
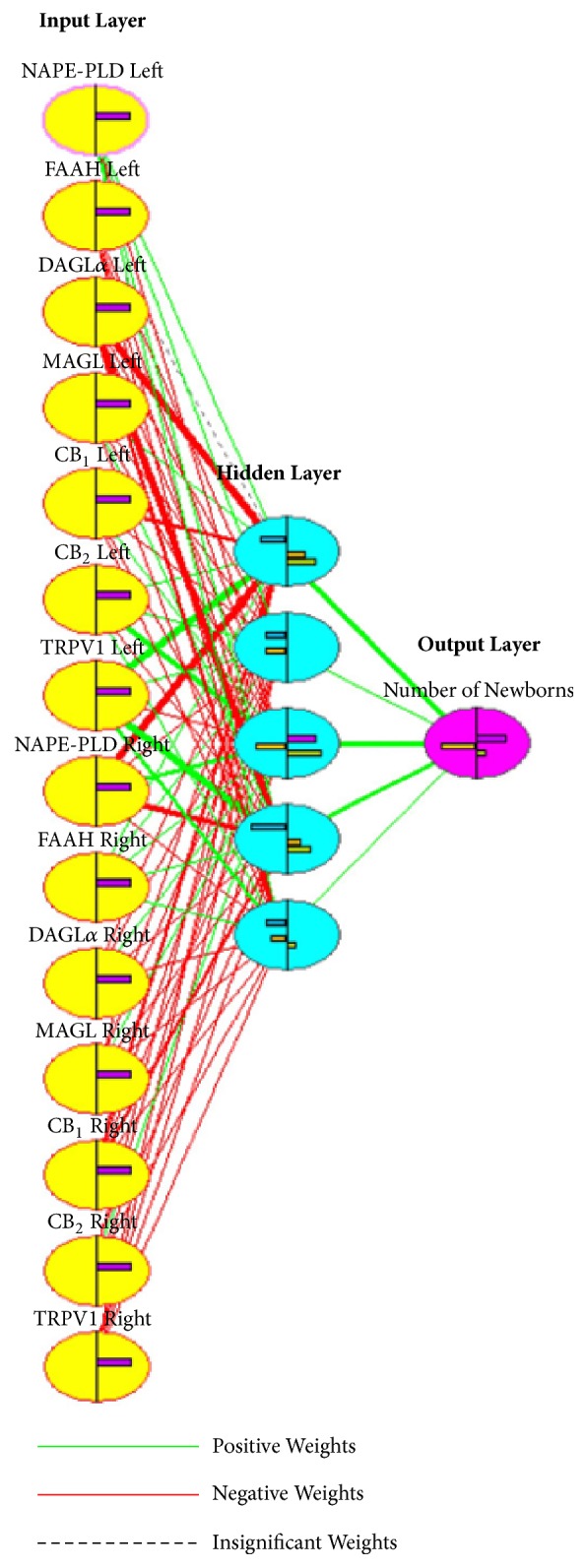
**Artificial Neural Network (ANN)**. Diagram showing the structure of the ANN made using the single ECS gene expression levels recorded in left and right testes versus the mean reproductive outcomes. The yellow circles indicate the input layer represented by the expression of every ECS target gene recorded in left and right testes of all the experimental animals (*n* = 35). The purple circle represents the output layer, that is, the mean reproductive outcomes. The light blue circles display the ANN hidden layer. Green, red, and dashed lines represent the edges of the network: links with a positive weight in the network are showed by the green lines, the red lines are of negative weights, and the dashed lines indicate insignificant connections. The thickness of the lines is directly proportional to the weights of the edges in the network architecture.

As a consequence, the relationship between ECS gene expression profiles and fertility to VAR experimentally induced pathologies was further investigated adopting a more sophisticated statistical method. To this aim, an artificial neural network (ANN) was developed (Figure 6). The ANN was built up by using as inputs 14 nodes containing ECS target gene expressions recorded in both testes of different experimental groups (CTRL and VAR). As outputs, the reproductive outcomes expressed as the mean number of new-borns were used, representing the biological outputs of the ANN. The hidden layer is the context of the network where the information is automatically processed in a blinded manner. In total, the ANN realized accounts of 75 edges connecting 20 nodes (Figure 6).

**Figure 7:**
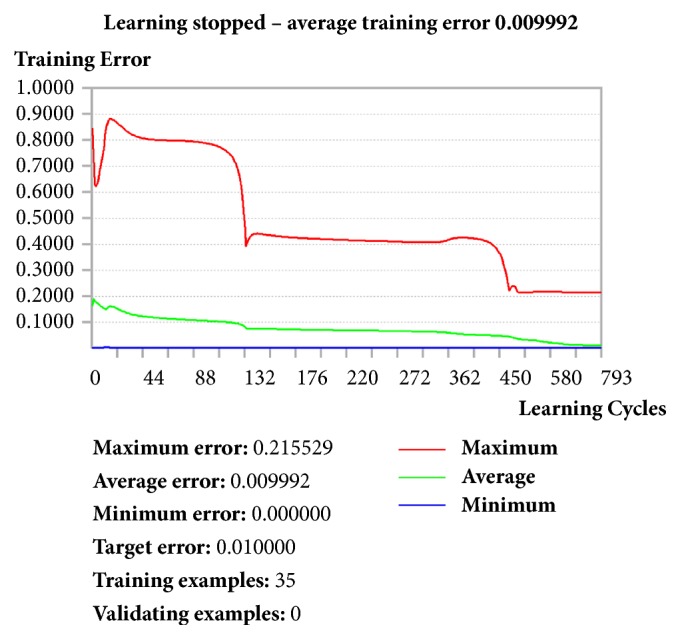
**ANN learning process.** The picture summarizes the ANN learning process automatically performed after the introduction of ECS gene expression levels (inputs) and mean reproductive outcomes (outputs). As shown in the first column of keys, this analysis was carried out by taking into account the datasets (paired inputs/outputs) obtained from all the experimental animals (*n* = 35) as training examples. During 793 learning cycles the ANN displayed a progressive decrease of the training errors that reached, at the end of the learning step, a minimum, maximum, and average prediction error of 0.000000, 0.215529, and 0.009992, respectively. Red line: maximum error. Green line: average error. Blue line: minimum error.

The system has been trained, through the backpropagation error algorithm [57], to use randomly identified ECS genes expression values as validating values, in order to assess the reliability of the network. The datasets (paired inputs/outputs) obtained from all the experimental animals (*n* = 35) were used as training examples, and the output value was generated after 793 learning cycles, with a progressive decrease of min, max and average error that reached, at the end of the learning process, 0.000000, 0.215529, and 0.009992, respectively (Figure 7).

As quality control, validating cycles were performed, in which five randomly selected datasets of the ANN were used as validating examples to test, at the end of the validating procedure, the validating error value. The network successfully completed the validating step, as indicated by the decline of the validating error which drops, at the end of the process, down below the 10% (Supplementary Fig.  1). As a result, by passing both the training and validating procedures, the ANN realized was able to develop a forecasting model of the reproductive outcome by using the ECS gene expression levels measured in both the left and right testes as inputs, with an average prediction error of 1% (0.009992).

Finally, when the training and validating procedures were concluded, it was possible to estimate the relative importance of all the single nodes, by considering the weights automatically attributed to each of them by the system. As a result, TRPV1 mRNA content in left testes was highlighted as the most relevant input in determining the output of the network, followed by DAGL*α* gene expression in left testes, and NAPE-PLD expression in right testes. The complete list of the input nodes in a decreasing order of importance is reported in Supplementary Fig.  2.

## 4. Discussion

Despite considerable advances in our understanding of the etiopathogenesis of varicocele, its relationship with fertility remains to be elucidated. Specific issues on the biological impact of varicocele upon testes are difficult to address in the absence of human tissues and/or targeted clinical studies. Therefore, animal models were used over the last thirty years to interrogate this major reproductive disease. In particular, the validated rat varicocele model offers a high translational value due to its capability of replicating several aspects of the human pathology, including alterations in testicular blood flow, spermatogenesis, endocrine functions, and immune response [53], as well as germ cell damage and apoptosis, hypoxia, oxidative stress, and heat stress [58].

Varicocele is known to potentially exert a detrimental effect on spermatogenesis [5, 7], although not all men affected by varicocele display semen defects [11, 13, 19] and fertility alterations [11, 19]. Yet, varicocele can be detected both in men with abnormal semen and in those with normal seminal parameters with no differences in their ability to father children [11]. Therefore, it is difficult to collect clinical information able to predict the reproductive impairment. Based on this evidence, the experimental varicocele animal model used in this study determined a high variability in terms of spermatogenesis quality, measured as Johnsen score values [55]. On the other hand, VAR animals with a high Johnsen score did not always show normal fertility, and those with a low Johnsen score does not always report fertility alterations, in keeping with human data showing that spermatogenic damage does not strictly relate to fertility outcomes [11, 13, 19]. These results support the concept that the evaluation of the testicular architecture* per se* cannot suitably explain the complex relationship between varicocele and fertility that are characterized by several pathogenic mechanisms resulting in different types/degrees of cell damages, spanning from DNA fragmentation [59] and genetic and epigenetic disorders [60], to various biochemical defects [61].

Since the conventional clinical investigations do not allow estimating the impact of varicocele on male fertility, the identification of molecular biomarkers represents a better way to improve the diagnostic efficacy. In this context, the present research suggests, for the first time, the possibility to candidate some ECS molecules as markers for predicting the impact of varicocele on fertility, thus strengthening the central role of this system in male reproductive physiology [23–25, 35–38]. Among different components of ECS, a crucial role in varicocele seems to be exerted by TRPV1. Indeed, the surgical induction of varicocele determined a significant down-regulation of TRPV1 gene expression in left testes, without affecting gene expression of any other ECS component under study. Incidentally, TRPV1 is a nonselective cation channel that acts as a polymodal sensor and molecular integrator of different noxious stimuli, including heat stress [62, 63] and oxidative stress [64, 65] that represent two major hallmarks of varicocele pathogenesis [1, 6, 10]. The activation of TRPV1 is known to be associated with cell protection and apoptosis [66–68], and this ion channel seems to operate as an important protective pathway even at gonadal level. Indeed, testicular hyperthermia in TRPV1^–/–^ mice results in a much more rapid and massive germ cell depletion [69]. According to this, the results of the present research support the idea that downregulation of testicular TRPV1 expression may deprive the testis of a protective mechanism against the detrimental milieu induced by varicocele, reducing in turn the fertility potential. The correlation between testicular TRPV1 expression and fertility impairment in left testes seems to confirm this hypothesis. However, TRPV1 receptor is just a piece of an integrated system [70], where reciprocal correlations among ECS elements can play a major role. Thus, to better understand the possible mutual link between TRPV1 and other ECS components, additional statistical analyses were performed. A multiple regression model and an ANN were used to disclose the complexity of the reproductive pathways, where interactions among single elements on the same system are known to play a central role [71, 72]. In terms of model fitting, the multiple regression analysis clearly highlighted a marked improvement, although from a biological point of view it was still unable to define a function capable of describing and approximating the relationship between testicular ECS gene expression patterns and reproductive outcomes with a satisfactory degree of accuracy (multiple R = 0.693 and R^2^ = 0.480).

The only analytical model that was able to contextualize the testicular ECS gene expression to the complexity of the reproductive function in terms of fertility outcome was a more sophisticated approach represented by ANN. It should be recalled that ANNs are computer-based algorithms inspired by the architecture and behaviour of neurons in human brain [73, 74] that are already used in biology for a variety of complex issues such as the prediction of protein secondary and tertiary structures [75, 76] and forensic age prediction using DNA-methylation patterns [77]. To date, ANNs represent a new frontier to interpret biological data, in order to develop novel diagnostic tools and targeted gene therapies [78, 79]. Noteworthy, the ANN designed in this study successfully provided a tool capable of predicting the fertility outcomes of the experimental subjects with a very high predictivity (average prediction error ≤1%), starting from their testicular ECS gene expression profiles. In this context, testicular ECS gene expression profiles were used as molecular inputs, whereas reproductive outcomes were set as their relative biological outputs. The backpropagation error algorithm [57] was used to train the network until the average prediction error of the system reached a significant final value of 0.009992.

Besides representing a novel diagnostic tool to predict the fertility outcome in varicocele, ANN provides additional information that aids interpretation, for example, of the role of each ECS gene in the predictive process. Indeed, after the training and the validating procedures were completed, ANN was able to assess directly from its architecture the relative importance of all input nodes in determining the output of the system, by considering the weights automatically attributed during the learning process to each of their connections. Among all inputs, TRPV1 gene expression in left testes was confirmed to be the most relevant node of the network among the different component of ECS, although also DAGL*α* and NAPE-PLD gene expression in left and right testes, respectively, showed a substantial role in the network architecture. Therefore, it could be argued that the regulation of DAGL*α* and NAPE-PLD genes may in turn guarantee a balance of 2-AG and AEA levels, respectively, overall sustaining an appropriate “testicular endocannabinoid tone” for correct spermatogenesis progression, as reported in mouse germ cells [36, 38, 48].

The real power of such a statistical approach was the ability to process all information in parallel and to detect complex patterns among all variables. Indeed, the single ECS input nodes possessed different weights in the network architecture, and the ANN capacity to simultaneously process and integrate all their contributions as well as their reciprocal interactions allowed the development of a reliable predictive model by overcoming the analytical limits of the conventional statistical methods.

## 5. Conclusions

The present study provides an unprecedented interpretative approach to evaluate the effect of ECS testicular gene expression on fertility in a validated experimental model of rat varicocele. Here, a central role for TRPV1 expression in varicocele was demonstrated, even though further experimental studies are needed to better understand the function of this receptor in the etiopathogenesis of this complex disease. Another relevant result of this research seems the ability to provide an open access ANN that is able to predict the fertility outcome with an accuracy of 99%, by inserting the testicular expression patterns of the ECS genes from varicocele-affected rats. In conclusion, this study represents the first proof of concept to translate an innovative ANN approach to human patients suffering from varicocele, as well as extend its diagnostic value to ECS protein levels, thus increasing the clinical relevance of the results.

## Figures and Tables

**Table 1:** Primer sequences used for ECS gene expression profiling.

**Target**	**Forward primer**	**Reverse primer**	**Efficiency**
NAPE-PLD	5′-TGTCCCGGGTTCCAAAGAGGAGC-3′	5′-ACCATCAGCGTCGCGTGTCC-3′	1.94
FAAH	5′-ATGGAAGTCCTCCAAGAGC-3′	5′-TAGAGCTTTCAGGCATAGCG-3′	1.95
DAGL*α*	5′-ATTCTCTCCTTCCTCCTGC-3′	5′-ATTTGGGCTTGGTGCTTCG-3′	1.91
MAGL	5′-ATGTTGAAGAGGCTGGACATGC-3′	5′-ATGCAGATTCCGGATTGGC-3′	1.94
CB_1_	5′-TTCCACCGTAAAGACAGCCC-3′	5′-TCCACATCAGGCAAAAGGCC-3′	1.96
CB_2_	5′-TTGACCGATACCTATGTCTGTGC-3′	5′-TGCTTTCCAGAGGACATACCC-3′	1.92
TRPV1	5′-ATTGAACGGCGGAACATGACG-3′	5′-ATCTCTTCCAGCTTCAGCG-3′	1.97
GAPDH	5′-AGACAGCCGCATCTTCTTGT-3′	5′-CTTGCCGTGGGTAGAGTCAT-3′	1.99

**Table 2:** Univariate correlation among reproductive outcomes and ECS gene expression levels in left and right testes.

						**Left**							**Right**				
		**N. of pups**	**NAPE-PLD**	**FAAH**	**DAGL**α	**MAGL**	**CB**_**1**_	**CB**_**2**_	**TRPV1**	**NAPE-PLD**	**FAAH**	**DAGL**α	**MAGL**	**CB**_**1**_	**CB**_**2**_	**TRPV1**	

	**N. of pups**		0.912	0.999	0.364	0.160	0.183	0.199	**0.023**	0.588	0.838	0.695	0.930	0.447	0.781	0.622	
	**NAPE-PLD**	-0.019		^**a**^ **0.003**	0.065	^**b**^ **1.2x10** ^**-5**^	0.787	0.602	0.207	^**c**^ **0.036**	0.348	0.053	0.195	0.693	0.232	0.384	
	**FAAH**	0.0002	^**a**^ **0.494**		0.309	^**d**^ **0.0002**	^**e**^ **0.042**	0.075	0.841	0.886	^**f**^ **0.017**	0.067	0.162	0.652	^**g**^ **0.048**	0.0775	
	**DAGL**α	-0.158	0.315	0.177		0.384	^**h**^ **0.012**	^**i**^ **4.9x10** ^**-5**^	^**j**^ **0.023**	0.843	0.574	^**k**^ **0.019**	0.133	0.906	0.249	0.284	
**Left**	**MAGL**	-0.243	^**b**^ **0.668**	^**d**^ **0.591**	0.152		0.503	0.790	^**l**^ **0.005**	0.346	0.797	0.813	0.903	0.951	0.727	0.842	
	**CB**_**1**_	0.231	0.047	^**e**^ **0.346**	^**h**^ **0.418**	-0.117		^**m**^ **2.9x10** ^**-11**^	^**n**^ ** 5.9x10** ^**-6**^	0.090	0.402	0.071	0.813	0.0552	0.131	0.184	
	**CB**_**2**_	0.222	0.091	0.305	^**i**^ **0.630**	-0.047	^**m**^ **0.862**		^**o**^ **7.6x10** ^**-8**^	0.085	0.463	0.105	0.421	0.380	0.202	0.179	*p*
	**TRPV1**	**0.383**	-0.219	-0.035	^**j**^ **0.384**	^**l**^ **-0.462**	^**n**^ **0.684**	^**o**^ **0.767**		0.214	0.341	^**p**^ **0.049**	0.505	0.309	0.318	0.246	
	**NAPE-PLD**	-0.095	^**c**^ **0.356**	0.025	-0.035	0.164	-0.291	-0.296	-0.216		0.625	^**q**^ **0.027**	0.489	0.800	0.678	0.182	
	**FAAH**	0.036	0.163	^**f**^ **0.402**	0.098	-0.045	0.146	0.128	0.166	-0.085		^**r**^ **0.006**	0.725	0.105	^**s**^ **1.7x10** ^**-9**^	^**t**^ **9.6x10** ^**-8**^	
	**DAGL**α	0.069	0.330	0.313	^**k**^ **0.395**	0.041	0.309	0.279	^**p**^ **0.335**	^**q**^ **0.374**	^**r**^ **0.451**		0.294	^**u**^ **0.047**	^**v**^ **0.018**	0.062	
**Right**	**MAGL**	-0.015	-0.224	-0.242	-0.259	-0.021	-0.042	-0.140	-0.117	-0.121	-0.062	-0.182		^**w**^ **0.0001**	0.935	0.542	
	**CB**_**1**_	-0.133	-0.069	0.079	-0.021	-0.011	0.327	0.153	0.177	-0.044	0.278	^**u**^ **0.339**	^**w**^ **0.607**		^**x**^ **0.007**	^**y**^ **0.002**	
	**CB**_**2**_	-0.049	0.207	^**g**^ **0.336**	0.200	-0.061	0.260	0.221	0.174	-0.073	^**s**^ **0.820**	^**v**^ **0.399**	-0.014	^**x**^ **0.447**		^**z**^ **2.2x10** ^**-11**^	
	**TRPV1**	-0.086	0.152	0.302	0.186	-0.035	0.230	0.232	0.201	-0.231	^**t**^ **0.764**	0.319	0.107	^**y**^ **0.499**	^**z**^ **0.865**		

								*r*									

Lower-left corner: Pearson correlation *r *coefficient. Upper-right corner: correlation analysis *p* values. The underlined values express a significant correlation between TRPV1 expression in left testes and reproductive outcomes, as indicated by *r* and *p *coefficients. ^**a-z**^Superscripts indicate significant correlations either of single ECS gene expression level of left versus right testes or among different ECS gene expression levels recorded intra and inter testes. Bold data were considered statistically significant for *p* values < 0.05.

## Data Availability

All data used to support the findings of this study are available from the corresponding author upon request.

## Supplementary Materials

Supplementary Materials
** Supplementary Fig. 1 ANN validating process. **In this example the ANN was processed by using 5 randomly selected datasets as validating examples, whereas the remaining 30 served as training examples. Under this condition the ANN showed that the validating error settled definitely down below the 10% from approximately the cycle 3960. The validating error was steady up to the cycle 5800000 when the process was stopped. The red, green, blue, and orange lines display the dinamic trend of the maximum, average, minimum, and validating errors, respectively.** Supplementary Fig. 2** Relative estimated importance of all the input nodes used by the ANN. Once the training and validating procedures were completed, the network calculated the relative importance of each input node by considering the weights automatically attributed to their connections. The most relevant inputs identified by the network are plot in black bars, whereas the grey ones indicate the input nodes with a progressive lower relative importance.
