# Corrigendum to “Artificial Neural Network to Predict Varicocele Impact on Male Fertility through Testicular Endocannabinoid Gene Expression Profiles”

**DOI:** 10.1155/2020/2368941

**Published:** 2020-05-19

**Authors:** Davide Perruzza, Nicola Bernabò, Cinzia Rapino, Luca Valbonetti, Ilaria Falanga, Valentina Russo, Annunziata Mauro, Paolo Berardinelli, Liborio Stuppia, Mauro Maccarrone, Barbara Barboni

**Affiliations:** ^1^Faculty of Biosciences and Technology for Food, Agriculture and Environment, University of Teramo, 64100 Teramo, Italy; ^2^Faculty of Veterinary Medicine, Agriculture and Environment, University of Teramo, 64100 Teramo, Italy; ^3^Department of Psychological, Health and Territorial Sciences, School of Medicine and Health Sciences, University “G. d'Annunzio” of Chieti and Pescara, 66100 Chieti, Italy; ^4^Department of Medicine, Campus Bio-Medico University of Rome, 00128 Rome, Italy; ^5^European Center for Brain Research, IRCCS Santa Lucia Foundation, 00164 Rome, Italy

In the article titled “Artificial Neural Network to Predict Varicocele Impact on Male Fertility through Testicular Endocannabinoid Gene Expression Profiles” [1], information regarding the support of the Italian Ministry for Instruction, University and Research (MIUR), was omitted in the Acknowledgments section. The corrected section appears below:
