# Supplementary material for: Artificial Neural Network to Predict Varicocele Impact on Male Fertility through Testicular Endocannabinoid Gene Expression Profiles
Source: Biomed Res Int. 2018 Nov 13;2018:3591086. doi: 10.1155/2018/3591086 (PMC6258097; doi:10.1155/2018/3591086)
Supplement: Supplementary Materials — Supplementary Fig. 1 ANN validating process. In this example the ANN was processed by using 5 randomly selected datasets as validating examples, whereas the remaining 30 served as training examples. Under this condition the ANN showed that the validating error settled definitely down below the 10% from approximately the cycle 3960. The validating error was steady up to the cycle 5800000 when the process was stopped. The red, green, blue, and orange lines display the dinamic trend of the maximum, average, minimum, and validating errors, respectively. Supplementary Fig. 2 Relative estimated importance of all the input nodes used by the ANN. Once the training and validating procedures were completed, the network calculated the relative importance of each input node by considering the weights automatically attributed to their connections. The most relevant inputs identified by the network are plot in black bars, whereas the grey ones indicate the input nodes with a progressive lower relative importance. [file 3591086.f1.pdf]

Supplementary Materials

Artificial Neural Network to predict varicocele impact on male fertility through testicular endocannabinoid gene expression profiles

Authors:
Davide Perruzza[1#*], Nicola Bernabò[1#], Cinzia Rapino[2#], Luca Valbonetti[1], Ilaria Falanga[1], Valentina Russo[1], Annunziata Mauro[1], Paolo Berardinelli[1], Liborio Stuppia[3], Mauro Maccarrone[4,5§] and Barbara Barboni[1§]

Affiliations:
[1]Faculty of Biosciences and Technology for Food, Agriculture and Environment, University of Teramo, 64100 Teramo, Italy
[2]Faculty of Veterinary Medicine, Agriculture and Environment, University of Teramo, 64100 Teramo, Italy
[3]Department of Psychological, Health and Territorial Sciences, School of Medicine and Health Sciences, University “G. d'Annunzio” of Chieti and Pescara, 66100 Chieti, Italy
[4]Department of Medicine, Campus Bio-Medico University of Rome, 00128 Rome, Italy
[5]European Center for Brain Research, IRCCS Santa Lucia Foundation, 00164 Rome, Italy

[#]Equally first authors
[§]Equally senior authors

*Correspondence should be addressed to: dperruzza@unite.it

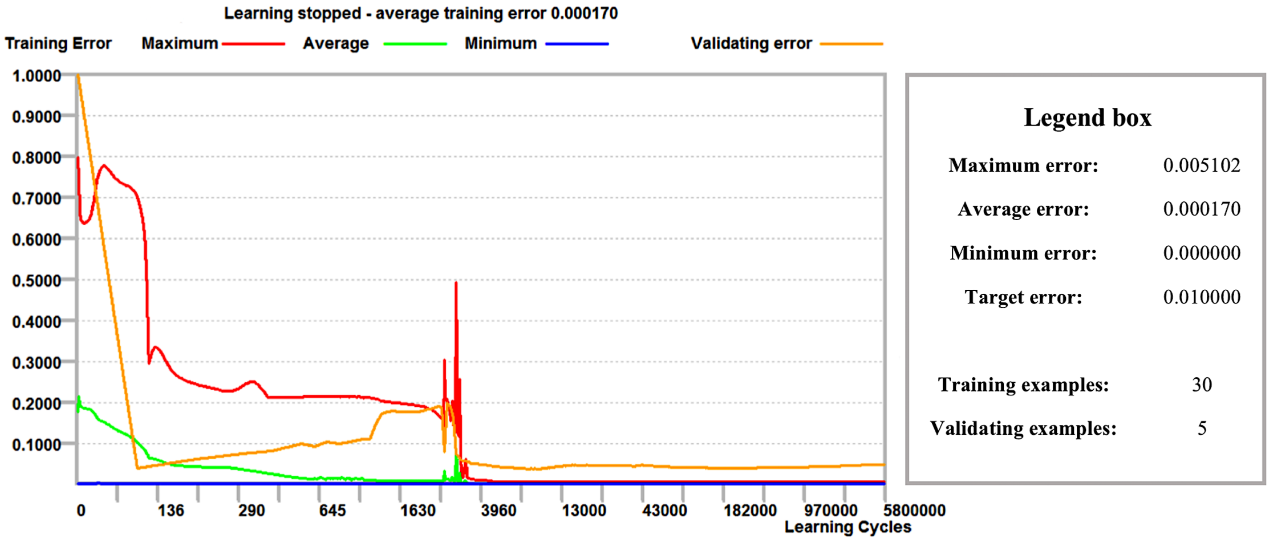

Supplementary Fig. 1 ANN validating process. In this example the ANN was processed by using 5 randomly selected datasets as validating examples, whereas the remaining 30 served as training examples. Under this condition the ANN showed that the validating error settled definitely down below the 10% from approximately the cycle 3960. The validating error was steady up to the cycle 5800000 when the process was stopped.
The red, green, blue, and orange lines display the dinamic trend of the maximum, average, minimum, and validating errors, respectively.

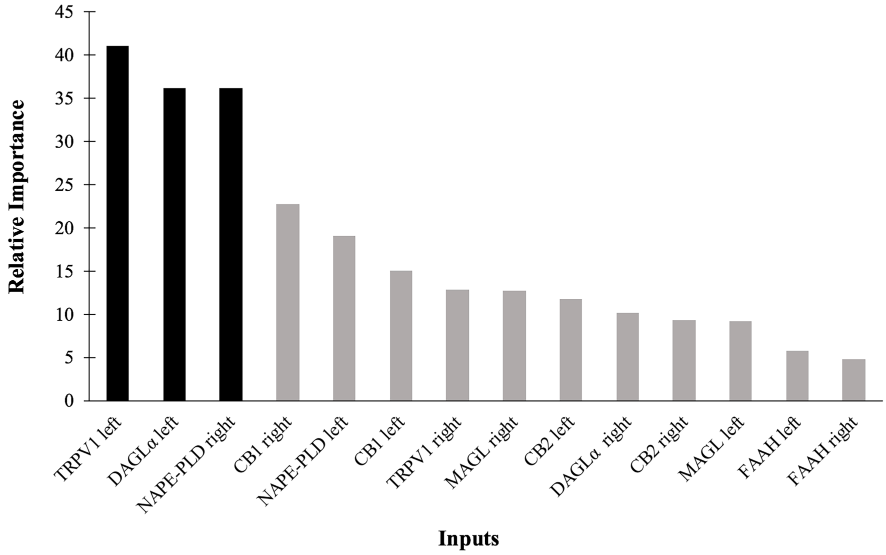

Supplementary Fig. 2 Relative estimated importance of all the input nodes used by the ANN. Once the training and validating procedures were completed, the network calculated the relative importance of each input node by considering the weights automatically attributed to their connections. The most relevant inputs identified by the network are plot in black bars, whereas the grey ones indicate the input nodes with a progressive lower relative importance.
